# Effect of extended egg quiescence and elevation in carbon dioxide on life history traits of *Aedes aegypti*

**DOI:** 10.1038/s41598-025-92193-4

**Published:** 2025-03-18

**Authors:** Sukritha Nalikkaramal, Sharon Rose Hill, Rickard Ignell

**Affiliations:** 1 Disease Vector Group, Department of Plant Protection Biology, Box 102, 234 56 Lomma, Alnarp Sweden; 2 Max Planck Center Next Generation Insect Chemical Ecology, Alnarp, Sweden

**Keywords:** *Aedes aegypti*, Carbon dioxide, Climate change, Egg quiescence, Feeding, Life-history, Metabolic reserves, Behavioural ecology, Climate-change ecology

## Abstract

Elevation in carbon dioxide is a global threat, driving anthropogenic climate change. How disease-vectoring mosquitoes respond to these changes is currently largely unknown. The dengue vector, *Aedes aegypti,* has adapted to urban environments, which are more affected by climatic changes, especially CO_2_. *Aedes aegypti* lay eggs around ephemeral water bodies that are prone to desiccation, with the pharate larvae possessing the ability to resist the desiccation, during which the permeability across the chorion is compromised. The study investigates the combined effects of elevated atmospheric CO_2_ and extended egg quiescence duration on life-history traits of immature and adult stages, including development rate, survival and size. Furthermore, we analysed the metabolic reserves of newly emerged females and whether mosquitoes display compensatory feeding in response to restricted reserves. Extended egg quiescence duration, combined with elevated CO_2_ level, differentially affected developmental duration and larval survival, with carry-over effects on adult metabolic reserves, size and survival. The interaction of elevated CO_2_ conditions and egg quiescence period differentially impact life-history traits of *Ae. aegypti*. The findings of this study provide evidential support for assertion that changing climatic conditions significantly impact survival and population dynamics, as well as feeding propensity, which directly affect the vectorial capacity of *Ae. aegypti*.

## Introduction

Global climate change, due to anthropogenic activities, is predicted to change the distribution of insects, including mosquitoes that vector disease, across spatial and temporal scales^1–3^. A major driver of this predicted change is the elevation in atmospheric carbon dioxide (CO_2_)^4^. Since the industrial revolution, the average CO_2_ level has almost doubled (420 ppm)^5^, and is predicted to increase to 600 ppm by 2050 and 1000 ppm by 2100^6^. The increase in global CO_2_ levels to date has led to a significant reduction in pH through acidification of oceans^7,8^ and freshwater bodies^9,10^ which adversely affects the residing organisms. Despite the growing concerns regarding the expanding geographic distribution of disease-vectoring mosquitoes, such as the primary vector of dengue, yellow fever, chikungunya and Zika, *Aedes aegypti*^11–15^, which can adapt to and occupy various ecological niches, little is known about how predicted elevation in CO_2_ levels will affect life history traits^2,16^. Such information may increase our understanding of the factors affecting population dynamics^17^ and feeding avidity, which intrinsically regulate vectorial capacity^18,19^.

Breeding water bodies of mosquitoes present both biotic and abiotic stresses that influence the survival and development of the immature stages^20^. For example, *Aedes aegypti* lay eggs in ephemeral water bodies that are prone to desiccation. As a result, the eggs have evolved to withstand periods of dormancy and desiccation, through egg quiescence^21,22^, the duration of which is regulated by environmental factors, such as temperature and humidity^20,23,24^. During the egg quiescence period, the larva depends on the maternal reserves for survival, and will hatch when favourable conditions arise^25^. Extended egg quiescence duration has been shown to affect the permeability of the chorion, as well as larval susceptibility to abiotic stressors, ultimately affecting adult fitness^26,27^. Weather conditions related to climate change, including warmer, wetter and drier conditions, have been demonstrated to affect larval hatching, survival and development, as well as adult fitness^28,29^. Moreover, water chemistry is known to affect life history traits, either directly^27,30–32^ or indirectly^33–35^. For example, elevated CO_2_ levels can reduce larval survival and increase developmental duration, as well as affect leaf litter decomposition^29^, although contradictory data has also been reported^34,35^. While elevation in CO_2_ has been shown to affect the physiology and behaviour of other freshwater-dwelling invertebrate life forms, including daphnia^8,36,37^, mussels^38,39^ and midget larvae^37^, their effect on mosquito life-history traits remain poorly understood.

This study investigated how extended pharate larval quiescence duration and elevated CO_2_ levels affect the life history traits of larval and adult stages of *Ae. aegypti*, including larval survival and developmental duration, as well as the carry-over effects on adult survival and body size. Additionally, the teneral metabolic reserves of females and their resource-feeding behaviour were assessed. The findings expand our understanding of how extended egg quiescence and elevation in CO_2_ levels interact to affect the development and survival of both immature and adult stages. These stress factors also affect the teneral reserves of female mosquitoes, regulating their feeding behaviour, which could have important implications for vectorial capacity.

## Results

### Developmental duration of immature stages and larval survival

**Fig. 1:**
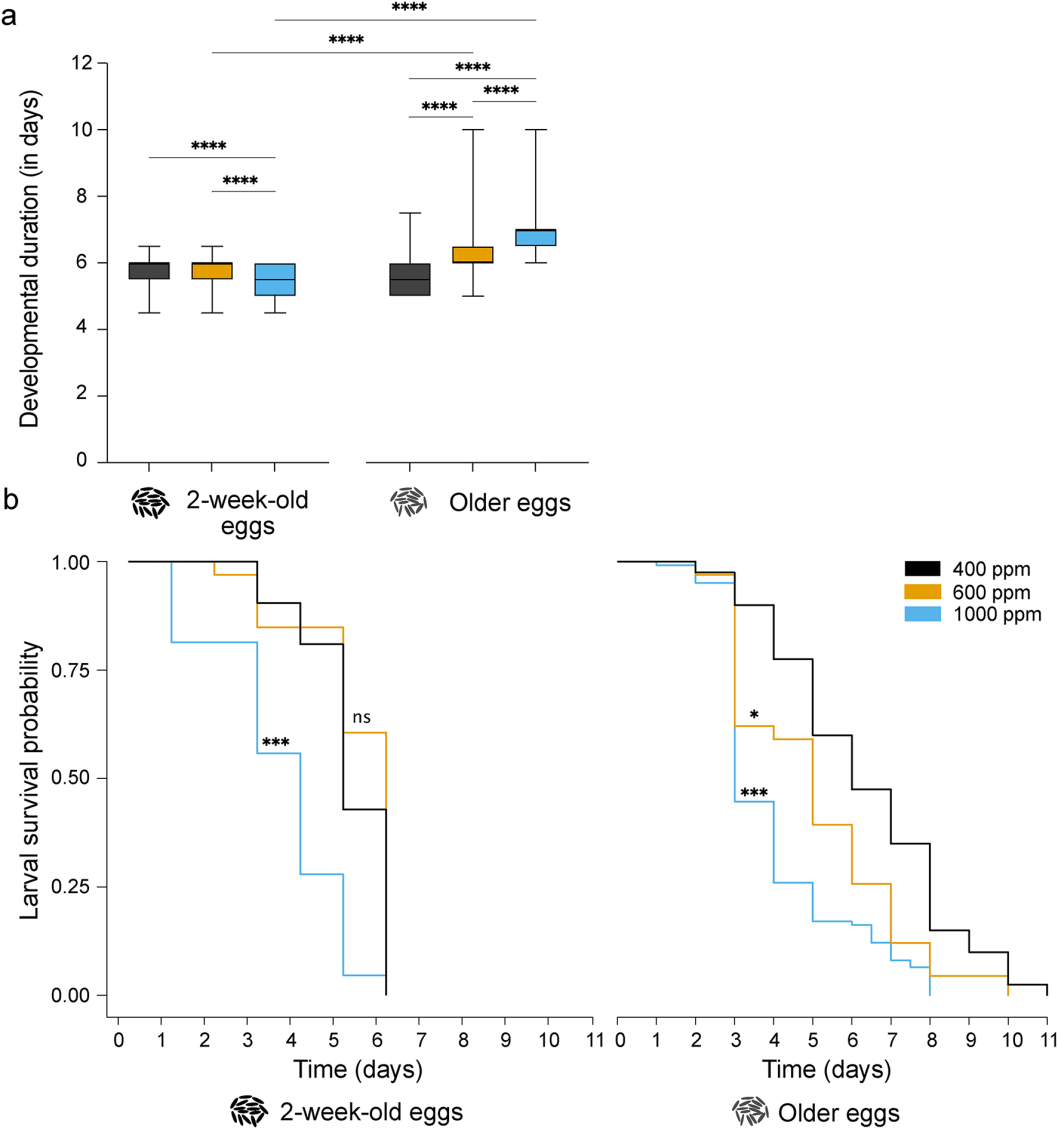
The effect of extended egg quiescence and elevated CO_2_ levels on immature stage development and survival. (**A**) Developmental duration of the immature stage from larvae to pupae. For comparison between groups, a Kruskal–Wallis test followed by Dunn’s multiple comparisons test was performed. The whiskers denote minimum to maximum, and asterisks indicate significant differences between the groups (N = 3, n = 300 larvae, p < 0.05). (**B**) Survival probability of the larvae originating from 2-week- and 3–6 months-old (older) eggs. The curves were analysed using a Cox regression model, followed by a log-rank *post-hoc* test using the ‘survival’ package (N = 3, n = 300 larvae, p < 0.05).

Elevation in CO_2_ level and the extent of egg quiescence significantly varied the developmental duration of immature stages from larvae to pupae (Kruskal–Wallis test; p < 0.0001; Fig. 1a). The developmental duration of immature stages, originating from 2-week-old eggs, was significantly reduced when reared under 1000 ppm CO_2_ condition (Fig. 1a). In contrast, the developmental duration of immature stages, originating from older eggs, increased significantly when reared under 600 ppm and 1000 ppm, compared to ambient CO_2_ condition (Fig. 1a).

The probability of larval survival varied significantly with an interaction between the level of CO_2_ (2-week-old eggs: Analysis of deviance, χ^2^ = 21.54, p < 0.001; older eggs: χ^2^ = 28.0, p < 0.0001, Fig. 1b) and extended egg quiescence (Analysis of deviance, χ^2^ = 1540.4, p < 0.0001, Fig. 1b). The survival probability of larvae originating from 2-week-old eggs reduced significantly at 1000 ppm CO_2_, whereas larvae reared under 600 ppm had a similar survival probability as those reared under ambient CO_2_ level (Fig. 1b). Similarly, the survival probability of larvae originating from older eggs, decreased when reared under 1000 ppm CO_2,_ as well as 600 ppm CO_2,_ compared to those reared under ambient CO_2_ condition (Fig. 1b).

### Effect on adult starvation tolerance and size

**Fig. 2:**
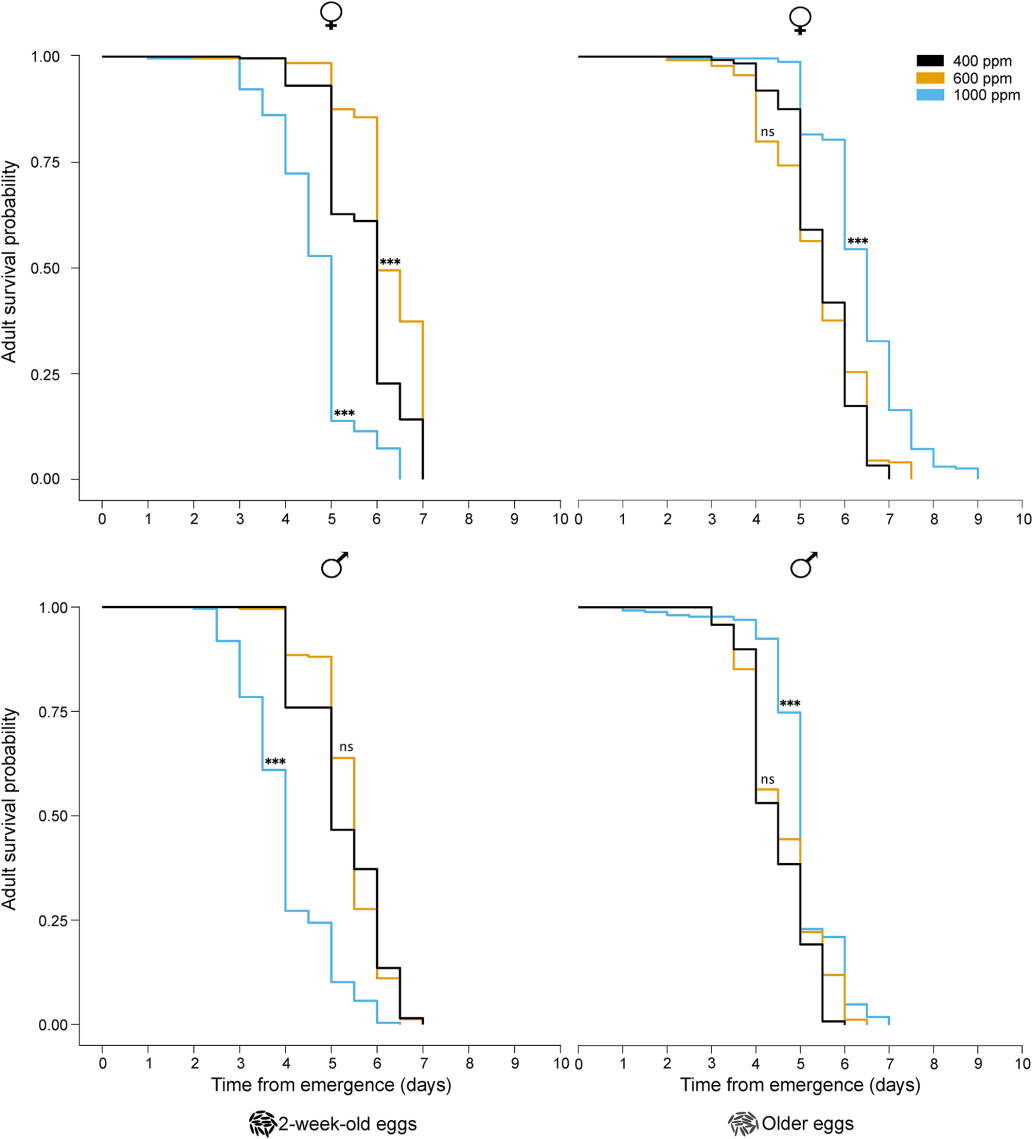
The interactive effect of extended quiescence and elevation in CO_2_ levels on the survival of adult *Aedes aegypti*. Survival probability curves of the adults are separated by sex and egg quiescence period. The curves were analysed using a mixed-effects Cox regression model, followed by a log-rank *post-hoc* test using the ‘survival’ package (n (per group) = 245–250, p < 0.05).

Adult starvation tolerance varied significantly with the increase in CO_2_ level (2-week-old eggs: Analysis of deviance, χ^2^ = 365.7, p < 0.0001; older eggs: Analysis of deviance, χ^2^ = 149.8, p < 0.0001; Fig. 2) and extended egg quiescence, with an interaction between the two factors (Analysis of deviance, χ^2^ = 150.5, p < 0.0001; Fig. 2). The starvation tolerance of females originating from 2-week-old eggs, increased at 600 ppm CO_2_, while starvation tolerance of both males and females reduced at 1000 ppm CO_2_, compared to adults reared under ambient CO_2_ conditions (Fig. 2), with significant differences observed between the sexes (Analysis of deviance, χ^2^ = 171.1; p < 0.0001; Fig. 2). In contrast, the starvation tolerance of females and males originating from older eggs was significantly different from each other (Analysis of deviance, χ^ 2^ = 405; p < 0.0001; Fig. 2), and increased when reared under 1000 ppm CO_2_, when compared to 600 ppm CO_2_ and ambient CO_2_ conditions (Fig. 2).

Adult size varied significantly with the increase in CO_2_ level and extended egg quiescence, (Kruskal–Wallis test; p < 0.0001; Supplementary Figure S1). The effect of elevated CO_2_ levels and extended egg quiescence was female-specific (Supplementary Figure S1), where the body size of females originating from 2-week-old eggs significantly decreased in response to an elevation in CO_2_ level (Supplementary Figure S1). Contrastingly, the size of females originating from older eggs did not differ across CO_2_ conditions and were significantly smaller than females originating from 2-week-old eggs, when reared under 600 ppm and ambient CO_2_ conditions (Supplementary Figure S1).

### Effect on total energy reserves

**Fig. 3:**
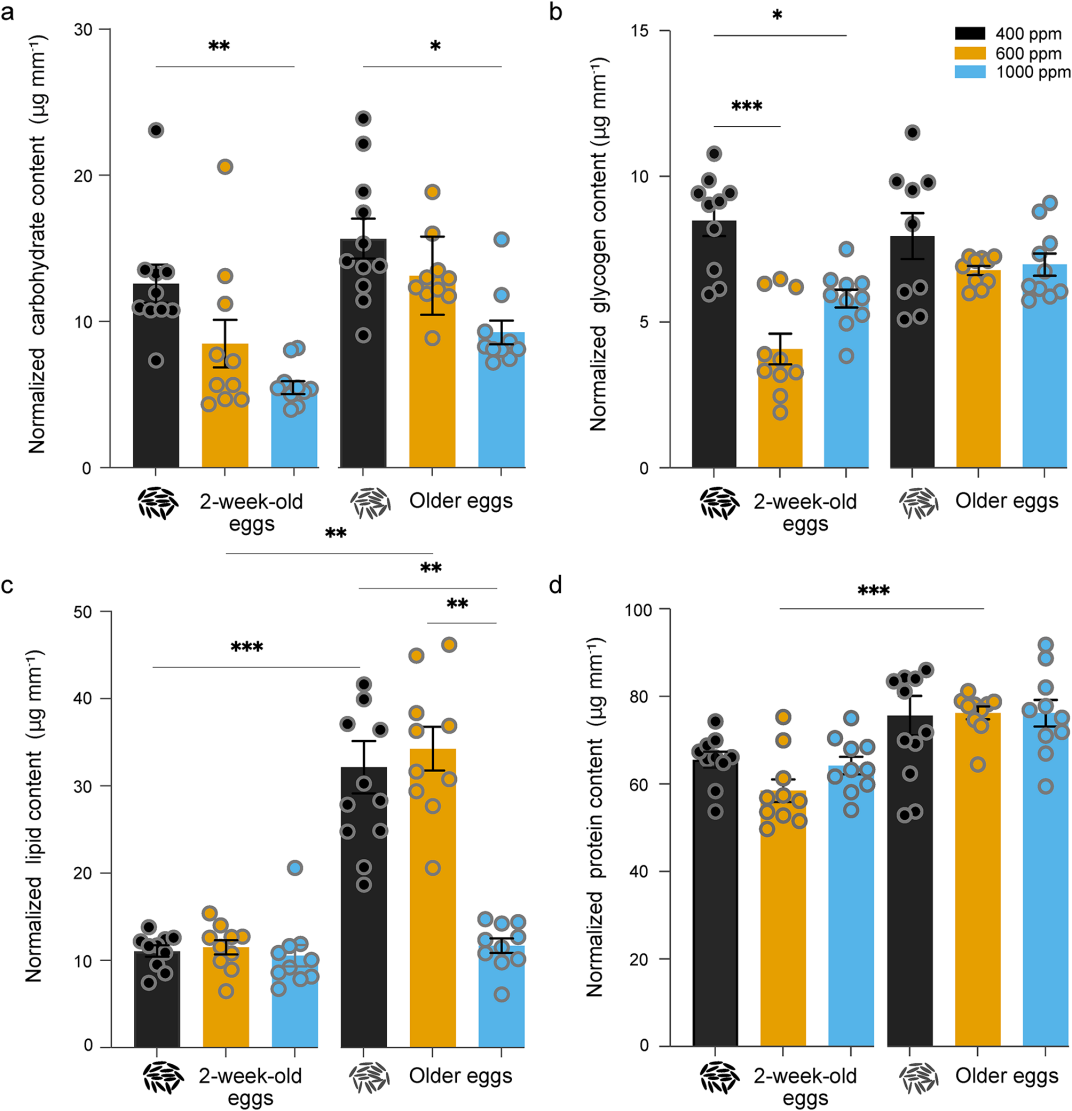
Metabolic reserves accumulated by teneral females in response to elevated CO_2_ levels and egg quiescence duration. The amount of soluble (**A**) carbohydrate, (**B**) glycogen, (**C**) lipid and (**D**) protein content normalized for body size. The error bars represent the standard error of the mean, and asterisks denote the significant differences between the groups. For comparison between groups, a Kruskal–Wallis test followed by Dunn’s multiple comparisons test was performed (n = 10, p < 0.05).

**Fig. 4:**
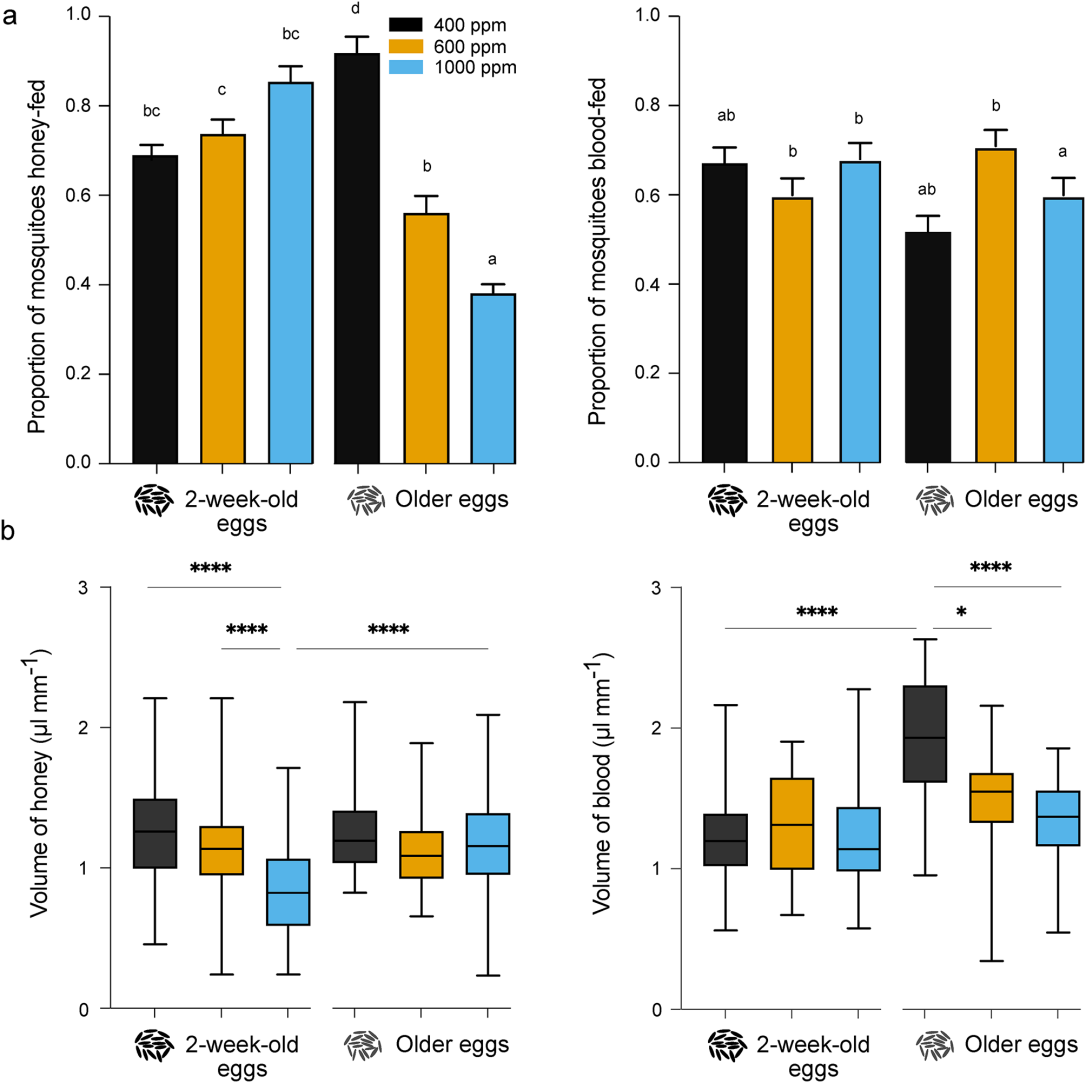
Differential feeding of female *Aedes aegypti* in response to elevated CO_2_ conditions and extended egg quiescence duration. (**A**) The proportion of teneral females that fed on honey (left) and blood (right) was differentially and significantly affected by extended egg quiescence duration and elevation in CO_2_ level. The bars represent mean (± SE) of proportion of females feeding and letters denote significant differences in pairwise comparisons using ‘emmeans’ Tukey method (n = 160–170 females, p < 0.05). (**B**) Volumetric analysis of imbibed honey (left) and blood (right) normalised for body size. For comparison between groups, a Kruskal–Wallis test followed by Dunn’s multiple comparisons test was performed. The whiskers denote the minimum to maximum values and asterisks indicate the significant differences between the groups (n = 50 females, p < 0.05).

Teneral metabolic reserves of individual females were analysed to quantify the total content of carbohydrates, glycogen, lipids and proteins accumulated during the aquatic stage in response to extended egg quiescence duration and elevated CO_2_ levels (Fig. 3). The soluble carbohydrate content varied significantly in response to both egg quiescence duration and CO_2_ conditions (Kruskal–Wallis test; p < 0.0001; Fig. 3a). The soluble carbohydrate content of females, irrespective of egg quiescence duration, was significantly lower when reared under 1000 ppm CO_2_ condition (Fig. 3a). The content of glycogen, which is a stored form of carbohydrate, differed significantly between females in response to egg quiescence duration and CO_2_ conditions (Kruskal–Wallis test; p = 0.0003; Fig. 3b). The glycogen content of females originating from 2-week-old eggs was significantly lower in response to elevated CO_2_ conditions, as opposed to the glycogen content of females originating from older eggs, that was not affected by CO_2_ conditions (Fig. 4b). The lipid content in females differed significantly in response to egg quiescence and CO_2_ conditions (Kruskal–Wallis test; p < 0.0001; Fig. 3c). The only significant pairwise comparisons were observed between females originating from older eggs, reared under 1000 ppm CO_2_ level, in which the lipid content was lower compared to the counterparts reared under 600 ppm and ambient CO_2_ conditions (Fig. 3c). The total protein content of females differed significantly in response to egg quiescence period and CO_2_ conditions (Kruskal–Wallis test; p < 0.0001; Fig. 3d). While the total protein content of females, originating from either egg quiescence conditions, remained similar across the CO_2_ conditions, females originating from older eggs had higher protein content compared to females originating from 2-week-old eggs when reared at 600 ppm CO_2_ (Fig. 3d).

### Feeding response of teneral females

No-choice feeding assays (Fig. 4A) were conducted to assess a potential compensatory feeding response by newly emerged female mosquitoes, as a consequence of the carry-over effects resulting from the stress caused by extended egg quiescence duration and elevated CO_2_ conditions. The proportion of females, which originated from 2-week-old eggs, that fed on either honey (Fig. 4a, left) or blood (Fig. 4a, right), was not significantly different between CO_2_ conditions. In contrast, females originating from older eggs fed significantly and proportionately less on honey with an increase in CO_2_ level (Fig. 4a, left). Moreover, the proportion of females emerging from older eggs, feeding on blood, was significantly reduced when reared under 1000 ppm CO_2_ compared to those reared under 600 ppm CO_2_ condition (Fig. 4a, right), as well as in comparisons with females emerging from 2-week-old eggs reared under 1000 ppm CO_2_ condition (Fig. 4a, right).

Colorimetric analysis was performed to quantify the volume that the females imbibed during the differential feeding on honey (Fig. 4b, left) and blood (Fig. 4b, right). Females originating from 2-week-old eggs reared at 1000 ppm CO_2_ imbibed a significantly lower volume of honey compared to females reared under 600 ppm and ambient CO_2_ conditions (Fig. 4b, left). In contrast, females originating from older eggs, irrespective of CO_2_ conditions, imbibed a similar volume of honey, while those reared from the younger eggs imbibed less at 1000 ppm CO_2_ (Fig. 4b, left). Females originating from both 2-week-old and older eggs, irrespective of CO_2_ conditions, generally imbibed a similar volume of blood (Fig. 4b, right). However, females originating from older eggs reared under ambient CO_2_ conditions, generally imbibed significantly higher volumes of blood compared to females reared under other CO_2_ conditions (Fig. 4b, right).

### Effect of artificial manipulation of water acidity

**Fig. 5:**
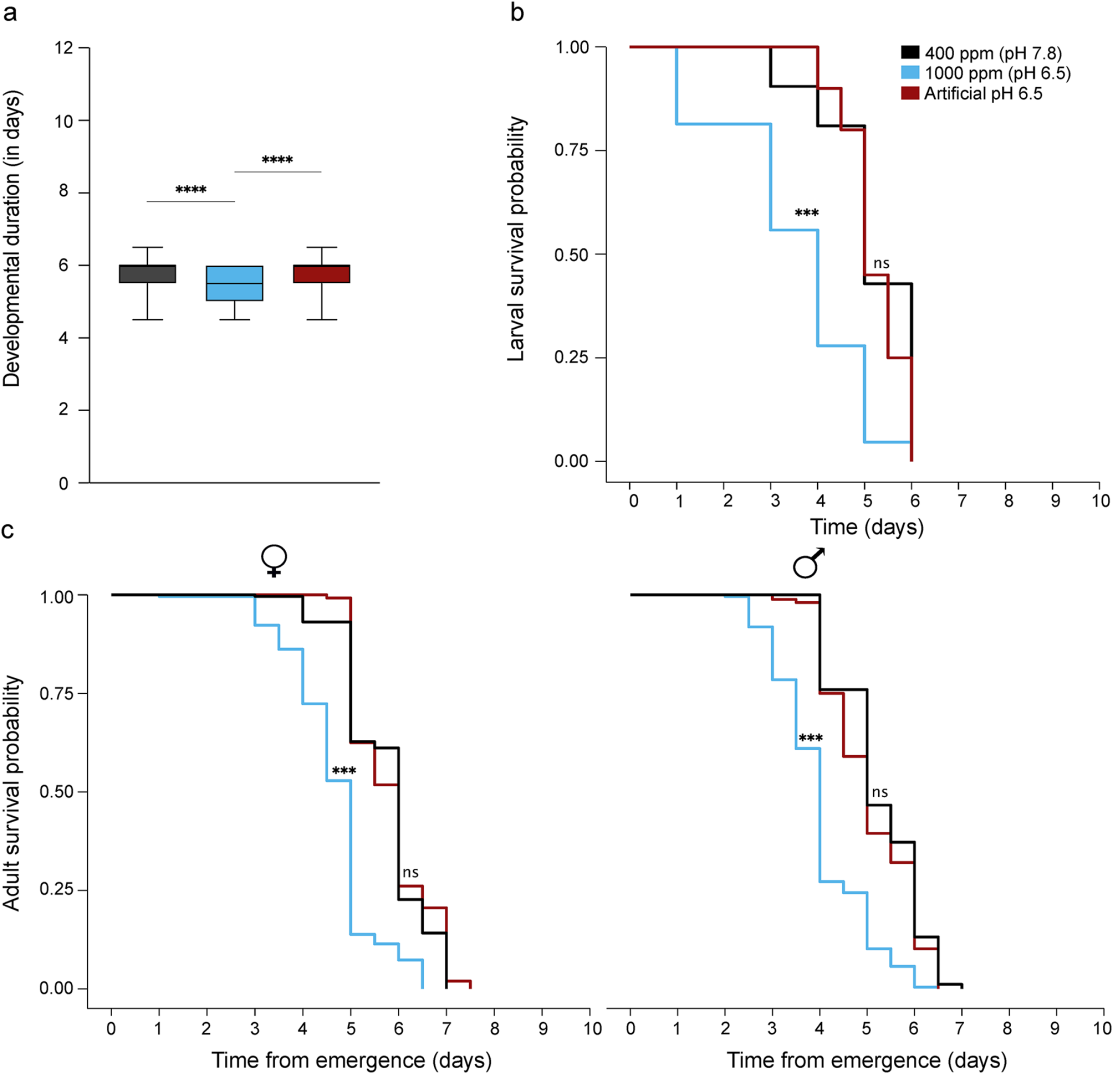
The effect of water acidification on the life history parameters of immature and adult stages of *Aedes aegypti*. (**A**) Developmental duration of the immature stage from larvae to pupae. For comparison between groups, a Kruskal–Wallis test followed by Dunn’s multiple comparisons test was performed. The whiskers denote minimum to maximum values, and asterisks indicate significant differences between the groups (N = 3, n = 300 larvae, p < 0.05). (**B**) Survival probability of the larvae originating from 2-week-old eggs reared under ambient and 1000 ppm CO_2_ conditions, as well as at pH 6.5. (**C**) Adult survival probability curves are separated by sex. The curves were analysed using a mixed-effect Cox regression model, followed by a log-rank *post-hoc* test using the ‘survival’ package (n = 245–250, p < 0.05). The data shown for ambient (pH 7.8) and 1000 ppm (pH 6.5) CO_2_ are the same as in Figs. 1 and 2.

The effect of artificially manipulated larval water pH was assessed on immature development duration, survival and adult starvation tolerance (Fig. 5). Artificial manipulation of water acidity did not have a significant effect on immature development duration (Kruskal–Wallis test; p = 0.90 Fig. 5a), larval survival (Analysis of deviance, χ^2^ = 1.05; p = 0.31 Fig. 5b) or adult starvation tolerance (Females: Analysis of deviance; χ^2^ = 1.78; p > 0.05, Males: Analysis of deviance; χ^2^ = 4.38; p > 0.05; Fig. 5c) when compared to ambient CO_2_ condition.

## Discussion

The atmospheric CO_2_ level is predicted to increase up to 1000 ppm within the next century. This increase, and even that predicted within a shorter period of time, significantly affects several life-history traits and feeding response of *Ae. aegypti*, an affect modulated by the extent of egg quiescence duration. While elevated CO_2_ conditions and extended egg quiescence duration negatively affected the aquatic stages, the carry-over effects to adults were differential. We present our findings below, in the context of what is known about the effects of CO_2_ and other climatic factors on mosquito life history traits, and what consequence this may have for population dynamics and vectorial capacity.

Elevation in CO_2_ directly and differentially affects larval development duration^33^ and survival, as well as adult survival and size, an effect that is dependent on the extent of egg quiescence duration. Whereas the negative effects of elevated CO_2_ levels observed in this study is in line with previous studies on other aquatic organisms^9,37,39,40^, the positive effect on adult survival has not been previously reported. When reared under elevated CO_2_ conditions, larvae that emerge from an extended egg quiescence duration had a delayed developmental duration. As pharate larvae depend on maternally-derived reserves, an extended egg quiescent duration leads to reduced energy reserves, increasing the vulnerability of larvae to sub-optimal or stressful conditions^27^. This limitation in energy reserves likely contribute to the significant reduction in survival of larvae emerging from an extended egg quiescence duration in response to elevated CO_2_ conditions, compared to those emerging from newer eggs, by negatively impacting the homeostasis^41^. Similar observations of higher mortality and delayed growth following exposure to elevated CO_2_ in copepods were attributed to additional energy demands^42^. To compensate for limited energy reserves upon emergence, larvae need to accumulate carbohydrate and lipid reserves, by feeding on, *e.g.*, detritus, to allow them to metamorphose into pupae^43^. This could explain the observed delay in developmental duration of larvae that emerged from an extended egg quiescence, at elevated CO_2_ conditions. Similar observations have been observed in mosquitoes and other aquatic organisms in response to various abiotic and biotic environmental stressors, including temperature, photoperiod and larval density^44–46^. An increased developmental time, not only increases foraging, thereby increasing the risk of predation, but also exposes the aquatic stages to habitat changes, such as drought^20^. In contrast to larvae emerging from extended egg quiescence duration, the larvae emerging from new eggs and reared at 1000 ppm CO_2_ had a significantly shorter development duration, which could be a defence strategy, in which larvae with higher maternal reserves pupate earlier and escape the stressful larval habitat^47^. Thus, larvae emerging from shorter or extended quiescence duration appear to have different adaptation strategies to environmental stress. These findings highlight the importance of considering the interactive effects of climatic factors, which play a critical role in influencing immature stage development and survival^47,48^, this study.

An elevation of CO_2_ in stagnant freshwater bodies cause physiological stress in aquatic organisms^39,49,50^, including mosquito larvae^35^, this study, through acidification of either the bodily fluids or the water^30,51^, or hypercapnia^49,52^. While elevation in CO_2_ can cause weak acidification in freshwater ecosystems^10^, water acidification in the absence of CO_2_ had no effect on the life history parameters of *Ae. aegypti* [this study], similar to what has been observed in *Daphnia*^9^ and freshwater zooplankton^36^. Hypercapnia-induced narcotic effects and associated effects on survival have been studied in other invertebrates^49,53^ and fish^54^. The mode of action of CO_2_, elevated to levels that reflect predicted changes, as used in this study, and how these affect the observed life history parameters, is unclear and requires further study.

Larval environmental parameters dictate the carry-over effects to emerging adults^55–58^, with significant effects on survival, size and reproductive success, as well as vectoral capacity^59^. Elevated CO_2_ levels during the aquatic phase significantly and differentially affected the survival and size of emerging males and females, an affect modulated by the interaction between egg quiescence duration and larval development duration. Similar interactive effects of environmental parameters with other abiotic stressors have been reported across other mosquito species^47,60^. The seemingly counterintuitive higher starvation tolerance in adults, following rearing at elevated CO_2_ conditions, could be indicative of metabolic priming, *i.e.*, adults emerging from stressful larval environments display anticipatory priming on their metabolic reserves^61,62^, which requires further investigation. We hypothesise that this metabolic priming likely obscures the correlation between metabolic reserves in teneral adults and survival, with body size having no significant effect.

Teneral mosquitoes differentially metabolise lipids or glycogen into carbohydrates depending on experienced egg quiescence period and CO_2_ conditions, but the low levels of accumulated reserves are not offset by compensatory feeding on either honey or blood. The glycogen and lipid content in teneral females originating from 2-week-old or older eggs, respectively, was significantly lower at elevated CO_2_ levels. Teneral females that originated from different egg quiescence periods appear to employ different metabolic strategies to cope with environmental stress, likely regulated at the metabolic enzyme activity level^62^, which requires further investigation. Similar to lipids and glycogen, the carbohydrate content in females was significantly low in response to the two stress factors, suggesting low energy reserves for locomotion during non-feeding periods^63^. While an expected response to this would be an increased compensatory feeding on either honey or blood, which is often used as an energy resource by nutritionally deprived mosquitoes^20^ the opposite was demonstrated, likely due to metabolic priming during the larval stage. Alternatively, nutritionally deprived mosquitoes may not be sufficiently motivated to spend energy to seek energy under the current conditions.

The vectorial capacity of mosquitoes hinges directly on the life history traits of the aquatic and adult stages, as well as the propensity of adult females to feed on human hosts. A slight reduction in development duration and survival in response to climatic change, although subtle, may have a significant and differential effect on mosquito population dynamics, which needs to be considered in future models. Moreover, these models need to take into consideration the effect of metabolic priming on starvation tolerance, and how elevated CO_2_ in combination with other climatic factors affect feeding patterns. Future semi-field and field studies will be required to further elucidate the effects of elevated CO_2_ on the life history trait of disease vectoring mosquitoes.

## Materials and methods

### Rearing of Aedes aegypti

For colony maintenance, *Ae. aegypti* (Rockefeller) were reared at 27 ± 2 °C and 65 ± 5% relative humidity, and a 12 h: 12 h light: dark cycle. Adult mosquitoes were provided ad libitum access to 10% sucrose solution, and females were allowed access to sheep blood (Håtunalab AB, Bro, Sweden), in a 1.5 ml reservoir covered with a collagen membrane using a membrane feeding system (Hemotek Ltd, Blackburn, UK), for egg production. Blood-fed females were given access to a wet conical filter paper placed above plastic cups filled with distilled water. Eggs laid on filter paper until 48 h were collected, labelled and stored in the rearing chamber until further use.

### Carbon dioxide acclimatisation

For the experiments, three high-precision climate chambers (ca 11.5 m^2^ with a free height of 2.3 m) were used, in which temperature, humidity and light were maintained as in the main rearing. The CO_2_ concentration in the chambers was set to ambient (ca. 400 ppm), 600 ppm and 1000 ppm, respectively, delivered through cylinders containing pure CO_2_ (Strandmöllen, Ljungby, Sweden), and regulated by the climate system. A filter paper containing age-controlled eggs (2-week and 3-to-6-month quiescent periods, respectively) from the main rearing was divided into approximately three equal parts and transferred to each of the climate chambers. The eggs were then placed in plastic larval trays (24 cm × 17.5 cm × 8 cm) filled with water (600 ml), previously acclimatised in the chambers for 48 h. A pinch of fish food (TetraMin® Flakes, Melle, Germany) was added to each tray to stimulate hatching^24^. The larvae that hatched within 18 h were divided into individual larval trays, with a density of 100 larvae in 600 ml of water. The larvae were fed fish food daily (1 mg larvae^-1^) to provide favourable conditions to maximise life-history parameters and reduce competition^47,64,65^, with food quantity adjusted to account for a reduction of larvae due to mortality. The water was changed every second day to control for microbial growth and accumulation of debris.

### Immature development and survival

Developmental duration, *i.e.,* the time from egg hatching to pupation, was assessed from observations done every 12 h, and differences between treatments analysed using a non-parametric test, Kruskal–Wallis test followed by Dunn’s multiple comparison test for select comparisons (GraphPad Prism, for Macbook 10.0.0 (131)). The pupae were collected every 12 h and transferred into small plastic cups with distilled water, and placed in Bugdorm cages (17.5 cm × 17.5 cm × 17.5 cm; Megaview Science Co., Ltd, Taichung, Taiwan) for further analysis of adult life history parameters. The larval survival probability was estimated by counting the number of live larvae every 12 h until all the larvae either pupated or died. Three independent replicates, each with 100 larvae, were conducted for each treatment and repeated thrice (Supplementary Figure S2). A mixed-effect Cox regression survival model was used to analyze the effect of elevated CO_2_ levels and egg quiescence on larval survival, with the replicate number and larval tray as fixed variables. A *post-hoc* test was then performed separately for the two egg quiescence periods with a log-rank test using the ‘survival’^53^ package in RStudio^54^.

### Adult starvation tolerance and size

Survival assays were conducted to assess adult starvation tolerance as a consequence of the metabolic reserves carried over from the immature stages. Emerging adult mosquitoes were provided access to distilled water, and the number of dead mosquitoes monitored every 12 h until all the mosquitoes in a cage were dead. To limit competition, each cage contained not more than 50 adult mosquitoes. A mixed-effect Cox regression survival model was used to analyze the effect of elevated CO_2_ levels and egg quiescence on adult survival with replicate number and cage as the fixed variable. A *post-hoc* test was then performed separately for the egg quiescence period and sexes with a log-rank test using the ‘survival’^66^ package in RStudio. For adult body size, the right wing of individual male and female mosquitoes was dissected under a stereomicroscope, and the distance from the axillary incision to the apical margin, excluding the wing fringes^68^, was measured using an ocular micrometre. For the analysis, Kruskal–Wallis test was performed followed by Dunn’s multiple comparison test for select comparisons (GraphPad Prism, for Macbook 10.0.0 (131).

### Estimation of teneral metabolic reserves

The teneral metabolic reserves were analysed by quantifying the carbohydrate, glycogen, lipid and protein content of individual adult female mosquitoes. For the analysis, adult females (up to 12 h post-emergence) were freeze-killed and stored in 2 ml Eppendorf tubes at -20 °C. The biochemical analysis was done according to van Handel’s calorimetric estimation methods modified by Foray et al.^69^. Protein analysis was performed according to Bradford’s method^70^, using the Bio-Rad Protein Assay Kit II (Bio-Rad Laboratories, Inc., Copenhagen, Denmark) with bovine serum albumin as a standard. Total carbohydrate and glycogen analyses were performed using anthrone (CAS: 90448, Sigma-Aldrich, Stockholm, Sweden) prepared in 95% sulphuric acid, with D-glucose (1 mg ml^-1^) as a standard^71^. Total lipid analysis was performed following a chloroform–methanol step, using vanillin (CAS: 121335, Sigma-Aldrich), prepared in 85% phosphoric acid, with olive oil (1 ml ml^-1^) as a standard^72^. The absorbance for the total carbohydrates (carbohydrate and glycogen), lipids and protein analyses were measured in 96 well plates at 625 nm, 525 nm and 595 nm, respectively, using a microplate reader (Multiskan **™** FC Microplate Photometer**,** Thermo Scientific**™**, Stockholm, Sweden). The content of carbohydrate, glycogen, lipid and protein was calculated based on comparisons with standard curves, adjusted for the dilution factor, and normalised for the mean wing size of the mosquito. For the experiment, ten females were randomly analysed for each treatment group. A comparison of medians was conducted with Kruskal–Wallis test followed by Dunn’s multiple comparisons test for select comparisons (GraphPad Prism, for Macbook 10.0.0 (131)).

### Feeding assays

No-choice feeding assays were conducted to correlate hypothesised compensatory feeding of females due to constraints posed by the metabolic reserves. Mosquitoes (24 h-to-48 h post-emergence) were aspirated into BugDorm cages in groups of 20-to-25 individuals per cage, and starved for 24 h with ad libitum access to water until 2 h prior to the start of the experiments. Experiments were conducted during the peak activity of the mosquitoes at Zeitgeber time 9–12^[73^, in the respective Biotron chambers. To assess the proportion of mosquitoes feeding and volume imbibed, all experiments were repeated six times, including two replicates of each egg batch. In addition, 20–25 mosquitoes from the same egg batch were provided access to water, and were used as controls for the volumetric analysis. After feeding, mosquitoes were carefully placed into 1.5 ml Eppendorf tubes and immediately frozen at-20 °C until further analysis. For the feeding proportion comparison, a generalised linear model with a binomial distribution followed by a *post-hoc* pairwise comparison with Bonferroni p value correction performed using the ‘emmeans’ package in RStudio^67^.

Honey (60% honey, prepared in distilled water) was used as the carbohydrate-rich source. To quantify the volume of honey imbibed, 1 mg ml^-1^ xylene cyanole (FF; CAS 2650–17-1; Sigma-Aldrich) was added, and fresh solutions prepared on the day of experiments. Mosquitoes were given access to the honey solution for 3 h. To quantify the volume imbibed, 230 µl of distilled water was added to the Eppendorf tubes containing the females provided with the honey solution. The tissues were then homogenised using a disposable pestle attached to a cordless motor (VWR®, Lund, Sweden), and then centrifuged at 6720 rcf for 10 min. The supernatant (200 µl) was transferred into individual wells of 96-well microplates (Sigma-Aldrich), and the absorbance measured at 620 nm using a spectrophotometer-based microplate reader. A standard curve was generated by preparing serial dilutions from 0.1 µl to 2.4 µl of 1 mg ml^-1^ xylene cyanol, and used to determine the volume of honey imbibed by each mosquito.

Sheep blood was used as the protein source, which was provided to the females for 1 h using the artificial membrane system described above. To quantify the volume of blood imbibed, the haemoglobinometry method^74^ was used. The abdomens of fed and unfed were dissected and homogenised in Drabkin’s reagent (prepared as detailed in^74^), followed by the addition of chloroform. The supernatant (200 µl) was pipetted into individual wells of 96-well microplates, and the absorbance measured at 540 nm using a spectrophotometer-based microplate reader. The absorbance of unfed mosquitoes was used as a control and subtracted from the absorbance of the blood-fed individuals. To determine the individual volume imbibed, a standard curve was generated using different volumes of blood. For the volumetric analysis, a comparison of medians was conducted with Kruskal–Wallis test followed by Dunn’s multiple comparisons test for select comparisons (GraphPad Prism).

### Artificial manipulation of water acidity

To determine the effect of change in acidity on life history parameters, the pH of the larval water used in this experiment was decreased to the level measured in the larval water maintained under 1000 ppm CO_2_, by the addition of 0.1 ml 0.1 N hydrochloric acid^8^; the pH was monitored throughout the experiment. Then, 2-week-old eggs were placed in the water and the life history traits of the emerging larvae and adults were assessed as described above.

## Supplementary Information

Supplementary Information.

## Data Availability

All data generated or analysed during this study are included in this published article.
