# Supplementary material for: Effect of extended egg quiescence and elevation in carbon dioxide on life history traits of Aedes aegypti
Source: Sci Rep. 2025 Mar 18;15:9310. doi: 10.1038/s41598-025-92193-4 (PMC11920594; doi:10.1038/s41598-025-92193-4)
Supplement: Supplementary file 1 — Supplementary Information. [file 41598_2025_92193_MOESM1_ESM.docx]

**Supplementary Information**

**
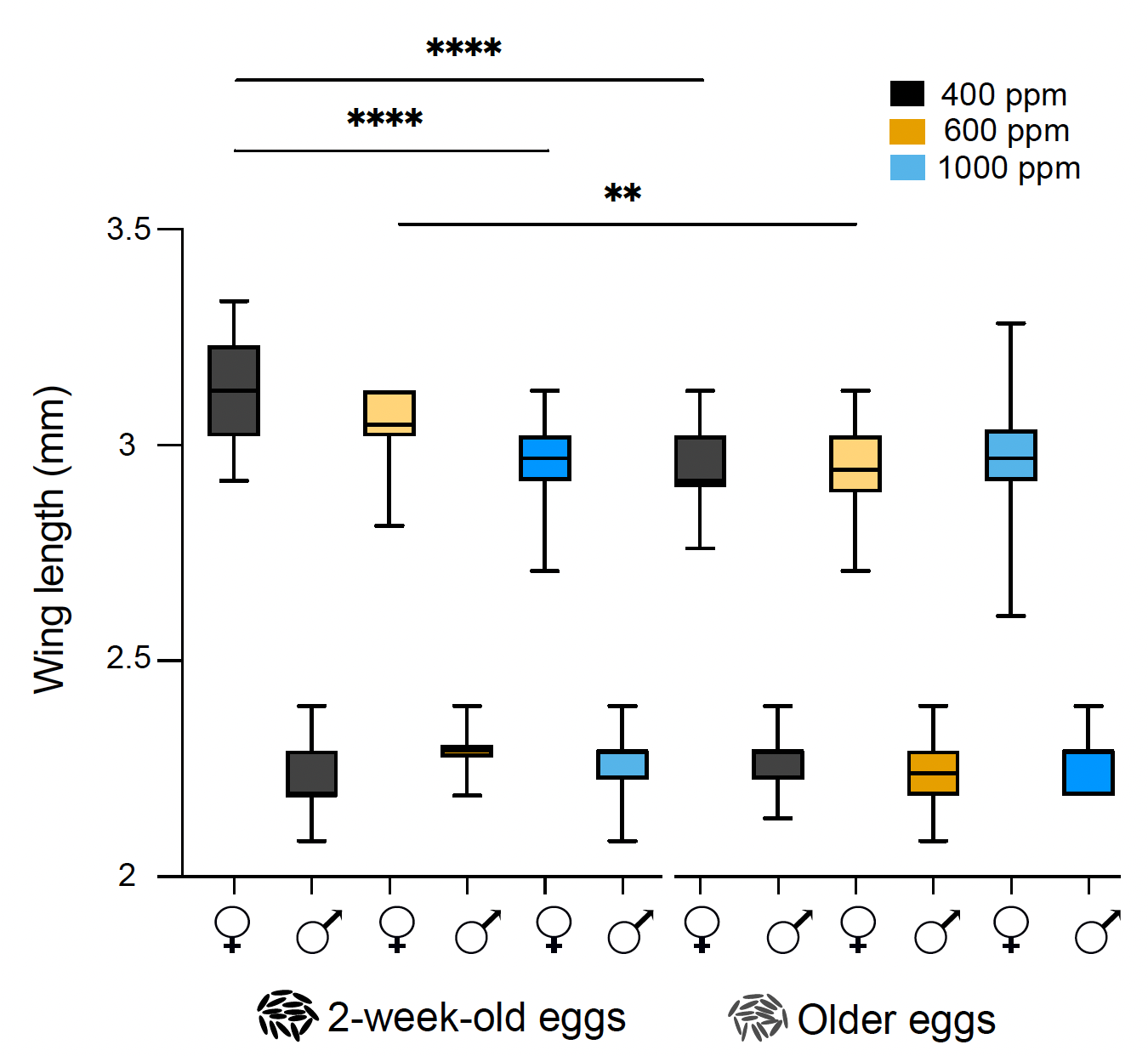
**

**Supplementary Information: Figure S1.** The interactive effect of elevated CO_2_ and egg quiescence period on adult body size. The wing size of both males and females were analysed using Kruskal-Wallis test followed by Dunn’s multiple comparison test for select comparisons. The whiskers denote the minimum to maximum values and asterisks indicate significant differences between the groups. (n= 30, p < 0.05).


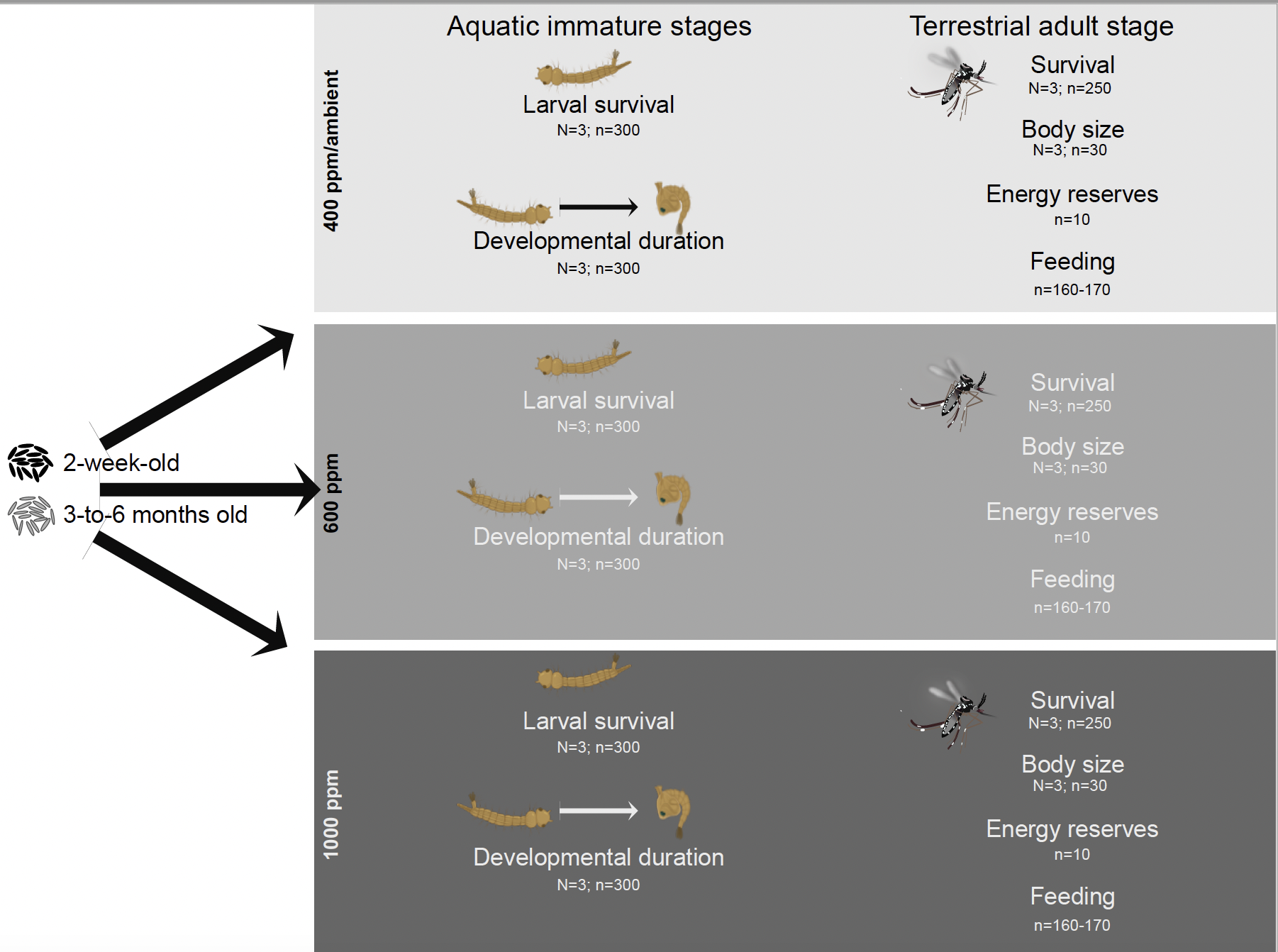


**Supplementary Information: Figure S2.** The graphical representation of the methodology used in this study to depict the sequence of experiments performed from aquatic immature stages to adult stages of *Aedes aegypti*. Larval and pupal illustrations: Created in BioRender. Sukritha, N. (2024) https://BioRender.com/u00u046 "
